# The extended hollowed mind: why foundational knowledge is indispensable in the age of AI

**DOI:** 10.3389/frai.2025.1719019

**Published:** 2025-12-11

**Authors:** Christian R. Klein, Reinhard Klein

**Affiliations:** 1 Clinic of Internal Medicine, University Hospital Bonn, Bonn, Germany; 2 Department of Computer Science, University of Bonn, Bonn, Germany

**Keywords:** artificial intelligence in education, cognitive sovereignty, sovereignty trap, cognitive atrophy, choice architecture, foundational knowledge

## Abstract

Generative artificial intelligence (AI) presents a fundamental duality for education: it simultaneously offers powerful cognitive extension while posing a significant risk of cognitive atrophy. This paper introduces the ‘hollowed mind’ as a conceptual framework to understand this risk—a state of dependency where the frictionless availability of AI-generated answers enables users to systematically bypass the effortful cognitive processes essential for deep learning. We argue this dynamic is driven by the ‘Sovereignty Trap’: a psychological mechanism where the AI’s authoritative competence tempts users to cede their own intellectual judgment, mistaking access to information for genuine ability. To substantiate this claim, we synthesize a multi-disciplinary body of evidence from cognitive science (e.g., dual-process theory, cognitive load), neurobiology (e.g., conflict-monitoring networks), and developmental psychology. We use this foundation to explain the widely documented ‘Expertise Duality’—why AI acts as a ‘leveler’ for novices but an ‘amplifier’ for experts. Moving beyond critique, this paper posits that the central challenge is one of environmental design, not user competence. We propose the ‘Fortified Mind’ as the pedagogical goal: a resilient internal architecture of indispensable knowledge and metacognitive skills required to achieve genuine ‘Cognitive Sovereignty’. Finally, we outline a forward-looking research agenda focused on redesigning AI tools from ‘answer engines’ into cognitive training environments that promote effortful engagement. Our work provides a robust conceptual guide for educators, researchers, and system designers, arguing that in the age of AI, the cultivation of fundamental knowledge is not just relevant, but more crucial than ever.

## Highlights

- *Conceptual Framework*: We introduce the Hollowed Mind as a risk state of cognitive under-engagement, the Sovereignty Trap as its underlying psychological mechanism, and the Fortified Mind as a desirable state of resilient cognitive sovereignty. Together, these concepts offer a testable framework for understanding how generative AI alters the balance between cognitive effort and automation.
- *Multi-Disciplinary Grounding*: We ground our framework in a deep synthesis of cognitive science (dual-process theory, cognitive load), neurobiology (conflict monitoring networks, neuroplasticity), and developmental psychology. This provides a robust biological and psychological basis for the argument that effortful, foundational knowledge construction is non-negotiable.
- *Explanation of the Expertise Duality*: We provide a theoretically grounded explanation for the widely observed “leveler vs. amplifier” effect of AI. By integrating the Expertise Reversal Effect and recent meta-analytic evidence, we argue that a user’s pre-existing knowledge is the critical determinant of whether AI partnership leads to dependency or genuine amplification.
- *A Forward-Looking Research Agenda*: Moving beyond critique, we propose a concrete, multi-pronged research agenda for the AI era. We outline key questions in system design, psychological mechanisms (e.g., longitudinal studies of cognitive atrophy), and socioeconomic implications, offering a clear roadmap for future empirical work.

## 1 Introduction: human–AI interaction between cognitive extension and the risk of cognitive atrophy

Generative artificial intelligence (AI), particularly in the form of Large Language Models (LLMs), has rapidly entered classrooms and everyday study practices, producing fluent explanations, code, and essays on demand. While comparisons are often made to earlier cognitive tools like calculators or search engines, this analogy is deceptive if not properly qualified. We propose a selection principle based on the type of cognitive process being offloaded: while the analogy to earlier tools is apt for procedural or retrieval-based offloading (often a rational trade-off), it becomes misleading for integrative reasoning. LLMs are qualitatively different: they do not merely automate discrete procedures or information retrieval but can directly perform tasks of integrative reasoning (Binz et al., 2025; Chen et al., 2021; Kosinski, 2024; Ouyang et al., 2022; Wei et al., 2022). This marks a fundamental shift in cognitive offloading, presenting not only unprecedented opportunities for learning but also a challenge that is unique in its nature and scale (Heersmink, 2024; Steyvers et al., 2025; Zhou et al., 2024). This fundamental shift creates a profound duality: The very capacity that leads generative AI to be celebrated as a powerful ‘cognitive extender’ (Clark, 2025; Clark and Chalmers, 1998; Hernández-Orallo, 2025)—its ability to automate the complex work of synthesis, argumentation, and evaluation—is precisely what allows users to bypass the mental effort required to build a resilient internal architecture for deep reasoning. Unlike with past tools, where the cognitive cost was arguably contained to discrete skills, the risk now is far more central. This gives rise to the central risk we term the ‘hollowed mind’: a state where the faculties for genuine Cognitive Sovereignty—the intellectual independence to govern, not just operate, our cognitive tools—are at risk of being inadvertently traded for the immediate gratification of an answer. This dynamic is potentially powered by the Sovereignty Trap—the predictable human tendency to choose the path of least cognitive resistance (using AI)—and it poses what may be one of the core design challenge for human-AI collaboration in education. It has been argued that a defining challenge of the 21st century is navigating profound uncertainty and conflicting goals (‘polytelia’)—a task requiring evaluative judgment rather than mere information processing (Funke, 2025). This very capacity is now recognized by industry as the most valued skill for the future of work (Digital Education Council, 2025)—and the one most acutely endangered by the temptation to outsource judgment to AI. The educational imperative, therefore, is not to prohibit the use of AI, but to design human–machine interactions that transform AI systems from simple “answer machines” into genuine frameworks for cultivating deep cognitive engagement. To ground this argument, the following sections will draw upon both emerging empirical evidence and established theoretical foundations—from cognitive neuroscience to developmental psychology—that offer a plausible account of the mechanisms behind the “hollowed mind.” This analysis aims to establish the rationale for the educational and technological recommendations outlined in the final part of this paper.

## 2 Psychological constraint: why analytical and deliberative reasoning is slow, effortful, and cannot be bypassed

### 2.1 The cognitive bypass: how AI design subverts the foundational principles of learning

Current AI systems are predominantly designed as oracles or assistants, a design paradigm that implicitly embodies the concept of AI as a “cognitive extender”—a seamless external augmentation of human cognition. Under this influential yet largely unchallenged paradigm, AI promises to liberate human minds by handling routine cognitive tasks, ostensibly freeing us for higher-order thinking: strategic oversight, ethical judgment, and creative innovation (Clark and Chalmers, 1998; Hernández-Orallo, 2025). While this paradigm is perfectly suited for instrumental tasks—a user fixing a one-off software bug—this paper argues that misapplying it to integrative learning contexts, where a student, professional, or lifelong learner seeks to build lasting internal knowledge, poses a significant risk. Consequently, educational discourse is now dominated by an emphasis on cultivating “fusion skills”—the ability to effectively prompt, supervise, and collaborate with AI systems. This attractive vision of AI as a ‘cognitive extender’, however, may rest on a potential misalignment with the principles of human cognition. It raises a profound and increasingly common question, especially from learners: “Why should I spend years building deep knowledge when an AI can deliver a perfect answer instantly? Why strive for expertise in a field where AI will always be superior?” This section contends that this question, while understandable, mistakes access for ability and convenience for cognition. The core issue may be the assumption that “just-in-time” access to information can replace the slow, deliberate process of knowledge internalization—a premise that is challenging to reconcile with the biological realities of learning. While an AI may master a domain overnight, humans require decades of sustained engagement to build the necessary cognitive scaffolding for genuine comprehension.

Psychologically, there are numerous theories and models that characterize these limitations of human cognition and learning. One of the most popular is the dual process theory (System 1/System 2) by Kahneman (2011) and Tversky and Kahneman (1974): Human thinking can be effectively modeled as the interplay of two fundamentally different systems. System 1 works quickly, automatically, intuitively, unconsciously, and emotionally. It is responsible for most of our everyday decisions and is based on heuristics (mental shortcuts). It is very efficient, but prone to systematic errors (biases). System 2 works slowly, consciously, logically, analytically, and requires effort. It is used for complex calculations, weighing arguments, and reviewing the suggestions of System 1. Its capacity is limited and it tires easily. Critical, deliberative thinking is the domain of System 2. It is (by definition) slow and laborious. The central challenge of rationality is to recognize when to distrust the fast but error-prone suggestions of System 1 and activate the laborious System 2. This process of “bypassing” System 1 is not possible; one can only consciously override it.

With the Type 1/Type 2 Processing model, Evans and Stanovich (2013) refined this fundamental distinction beyond the mere description of the systems to explain the mechanisms of rationality itself. Analogously, Type 1 processing is fast, autonomous, and does not require working memory capacity. Type 2 processing is slow, laborious, and requires working memory capacity. Stanovich argues that the ability to interrupt and override Type 1 processing when necessary is a core feature of rationality and higher intelligence. Analytical thinking (Type 2) is an active, strenuous intervention that does not happen on its own. According to Evans and Stanovich (2013), the effortless Type 1 process is the “default setting of the brain.”

The observation that analytical and deliberative thinking is slow and “effortful” is also explained by the Cognitive Load Theory (CLT) by Sweller (1988): CLT posits that human cognitive architecture is constrained by a severely limited working memory that can only process a few novel elements at once. Every piece of information we process generates a “cognitive load,” which consists of three types: the intrinsic load, which describes the inherent difficulty of the topic itself; extraneous load, which arises from the way information is presented (e.g., a confusing website layout, confusing language); and germane load, i.e., the effort expended on actual, deep learning and the construction of mental schemata. CLT explains why analytical thinking is exhausting: When extraneous load is high (e.g., from a superficially credible but misleading website), the limited resources of working memory are depleted. There is no capacity left for germane load—i.e., the slow, critical examination of facts. This process cannot be bypassed; when the “tank” is empty, one resorts to the simplest heuristics (System 1).

The current AI design paradigm interacts with this dynamic. By providing instantly plausible answers, these tools cater directly to our preference for low-effort System 1 thinking, allowing us to systematically bypass the rigorous engagement of System 2—the very process required for deep learning and knowledge internalization. The very goal of learning is to build complex knowledge structures, or schemas, in our vast long-term memory, which allows us to treat intricate concepts as single elements, thus circumventing the limits of working memory. The current AI “answer engine” paradigm bypasses learning by eliminating the need for germane load. By providing polished answers, it removes the necessary precursors for the cognitive work—such as cognitive dissonance or the need to resolve uncertainty—that are known to trigger schema construction processes like organizing, integrating, and abstracting. This act of cognitive outsourcing is arguably not a shortcut to knowledge; it risks being tantamount to the removal of core aspects of the learning process itself. This makes the effortful engagement of germane load a fundamental psychological prerequisite for building a fortified, rather than hollowed, mind (Sweller et al., 2011).

### 2.2 Neural correlates of heuristic and deliberative thinking

The distinction between a fast, heuristic thinking system and a slow, deliberative thinking system is deeply rooted in the functional architecture of the human brain: it is equipped with a robust conflict detection system, the main player of which is the anterior cingulate cortex (ACC). This system is activated whenever an intuitive, heuristic response (Type 1) contradicts the logical or statistical rules of a problem. However, whether this conflict is resolved in favor of logical analysis (Type 2) depends on the activation of another network: the cognitive control system in the lateral prefrontal cortex (LPFC), especially on the right brain. Early functional Magnetic Resonance Imaging (fMRI) studies by Goel et al. (1998) on the so-called “belief bias” were groundbreaking in this regard. They showed that the right LPFC was always recruited when subjects had to actively suppress the intuitive, belief-based response in order to arrive at a logically correct but implausible conclusion (Goel et al., 1998; Goel and Dolan, 2003).

Building on this evidence of prefrontal engagement during cognitive conflict, studies by Neys et al. (2008) investigated the process even more directly. Their seminal discovery was that the ACC showed increased activity even when subjects gave the incorrect, heuristic answer. This is crucial evidence: the brain recognizes the conflict on a subconscious level, even if the conscious, effortful Type 2 system is not activated strongly enough to override the intuitive answer. The “cognitive miser” therefore fails not because it fails to notice the problem, but because it fails to muster the cognitive effort necessary to solve it. Recent research confirms the robustness of this conflict recognition mechanism, even if the exact temporal processes are more complex than originally assumed (Bago and De Neys, 2017).

Within the context of AI and education, this is exactly where the current AI paradigm for learning reveals a key neurobiological vulnerability. True knowledge acquisition should not be considered as passive storage of facts, but an active process of overcoming cognitive dissonance—the moment when new information challenges established, often intuitive ideas. This process of “unlearning” requires the activation of the ACC-LPFC network. The brain must recognize the conflict and then do the hard work of restructuring existing mental models.

Today’s AI systems are optimized for maximum cognitive ease and smoothness. They provide immediate, linguistically fluent, and plausible-sounding answers. The neurobiological risk of this design is not that it systematically bypasses conflict recognition by the ACC entirely. Rather, it weakens the entire loop of conflict-driven learning through a two-fold challenge: First, the high plausibility of AI outputs may generate a weaker or more subtle conflict signal from the ACC. If an answer does not raise any obvious contradictions and minimizes the user’s cognitive effort, the threshold for the ACC to be robustly alerted may not be reached. Second, and more importantly, even if a conflict is detected, a user with low domain knowledge lacks the necessary schemas to effectively act upon that signal. Consequently, the prefrontal cortex is not recruited to critically examine the answer and compare it with existing knowledge. This creates a vicious cycle: low engagement prevents the building of the very domain knowledge that would enable future critical evaluation. Since neural circuits that are chronically under-stimulated become less efficient—a core principle of neuroplasticity known as “use it or lose it” (Lamprecht and LeDoux, 2004; Pascual-Leone et al., 2005), —excessive reliance on AI systems that reduce the likelihood of both conflict detection and resolution carries the risk of under-training precisely those prefrontal networks that are crucial for critical thinking. This suggests that the very cognitive effort that AI promises to relieve us of may be precisely the effort our brains need in order to learn.

### 2.3 The developmental timeline

Human cognitive development unfolds in broadly predictable stages and is characterized by fundamental biological and temporal constraints on learning, a process that appears largely resistant to technological acceleration. Jean Piaget’s foundational framework demonstrates that children require approximately 18 years to progress through fundamental cognitive stages—from basic object permanence in infancy to abstract reasoning in adolescence (Inhelder and Piaget, 1958). Building on this, King and Kitchener’s (1994) Reflective Judgment Model provides crucial insights into when humans can meaningfully critique expert-level content. Their seven-stage model shows that pre-reflective stages (viewing knowledge as certain and handed down by authorities) dominate childhood. Only in quasi-reflective stages (typically emerging in mid-to-late adolescence) do individuals begin to recognize uncertainty and the constructed nature of knowledge, with full reflective judgment—the very capability essential for evaluating probabilistic, often biased AI outputs—developing in late adolescence and early adulthood at the earliest.

However, this broad timeline offers a clearer understanding of a more granular, evidence-based view to understand the specific capabilities needed for AI evaluation. Research on epistemic cognition—how individuals think about the nature of knowledge and knowing—reveals a more detailed progression.

- Middle School (approx. Ages 11–14): learners can begin to identify simple contradictions and question the absolute authority of a source. They might ask, “Is this AI answer true?” However, they typically struggle to evaluate the quality of evidence, recognize subtle bias, or understand the probabilistic nature of a generative model. Their evaluation remains focused on the output itself, not the process that created it (Kuhn, 2005).
- High School (approx. Ages 15–18): with the emergence of more robust metacognitive abilities, adolescents can begin to think about how they know things (Flavell, 1979). They become more capable of coordinating multiple, conflicting sources of evidence and understanding that “facts” can be framed to support different arguments. This is the critical period where the capacity for true critical evaluation—asking “Why was this answer generated?” or “What perspective is missing?”—can be cultivated.

This developmental trajectory would be an oversimplification to regard as monolithic; it is profoundly shaped by individual and cultural differences. Individual differences in intellectual curiosity, captured by the “Need for Cognition” construct, mean that some individuals will more readily engage in the effortful thinking required to develop these skills (Cacioppo and Petty, 1982). Furthermore, a learner’s pre-existing knowledge base within a domain drastically alters their ability to critically evaluate AI outputs in that area, creating significant variation in capability even among peers of the same age (Bransford et al., 2000; Brod, 2021; Chi et al., 1981; Choi et al., 2025; Ng et al., 2021; Wineburg and McGrew, 2017; Wineburg, 1991). Research on memory consolidation reveals that while initial encoding happens quickly, the process of transferring memories to the neocortex for stable, long-term storage—systems consolidation—requires weeks, months, or even years (Dudai, 2004; Frankland and Bontempi, 2005). This slow, sleep-dependent process provides the non-negotiable neurobiological basis for proven educational principles like the “spacing effect,” where distributed practice vastly outperforms cramming (Dunlosky et al., 2013; Rasch and Born, 2013). Unlike sensory systems that stabilize in early childhood, the prefrontal cortex maintains heightened plasticity until approximately age 20, with parvalbumin interneuron maturation occurring only at the onset of puberty, making adolescence a crucial and sensitive period for higher-order learning and cognitive development (Casey et al., 2000; Fuhrmann et al., 2015; Giedd, 2004). This protracted development creates a vulnerable window where AI dependency could disrupt the very neural circuits responsible for cognitive oversight of AI systems. This extended plasticity period may therefore help to explain why adolescents and young adults could be particularly susceptible to AI-induced cognitive atrophy. The prefrontal circuits responsible for metacognition, critical evaluation, and executive control are still consolidating during the exact developmental period when students increasingly rely on AI tools. Finally, it is crucial not to mistake technological familiarity for critical evaluation capability. The “digital native” concept is a concept that has been widely critiqued as a myth. Extensive research, most notably from the Stanford History Education Group, has repeatedly demonstrated that today’s adolescents and young adults, despite their digital fluency, often demonstrate significant difficulties in evaluating the credibility of online information. They are easily fooled by markers of superficial credibility and consistently fail to engage in simple but effective evaluation strategies like “lateral reading”—leaving a website to investigate the source itself (Breakstone et al., 2021; Wineburg and McGrew, 2017). This gap between perceived and actual competence underscores our central argument: the slow, psychological development of critical and reflective judgment is a primary bottleneck that persists, especially along the developmental timeline, and cannot be easily bypassed by mere exposure to or facility with technology.

## 3 Empirical evidence for the hollowed mind

The concept of the “hollowed mind” refers to the cognitive risks that arise when reliance on AI outputs replaces the effortful processes of slow, deep learning. While direct, long-term evidence on generative AI is inherently limited by the technology’s novelty, these risks are not merely theoretical. An emerging body of empirical research—from early studies on digital tools to large-scale experiments with LLMs—provides initial, real-world support for the hypothesized risk of the hollowed mind and reveals the Expertise Duality at the heart of AI-driven productivity.

### 3.1 Precursors to the hollowed mind: digital amnesia and navigational atrophy

The process of cognitive erosion through offloading is a well-documented phenomenon. While we argue that LLMs pose a unique threat by automating integrative reasoning, early studies on tools that offload information retrieval and procedural navigation provide crucial evidence for the underlying mechanisms of skill decay. The seminal ‘Google effect’ study (Sparrow et al., 2011), for instance, serves as a powerful precursor that illustrates how the brain reconfigures itself in response to external memory stores. They demonstrated that when individuals know information will be externally accessible, they exhibit what is termed ‘digital amnesia’—people remember less of the information itself and instead remember the location or pathway to access it. This shift from internalizing content to internalizing access pathways systematically reconfigures the mind to be a directory, not a generator of thought. This ‘Google effect’ goes beyond simply outsourcing memory. Subsequent research demonstrates a more subtle consequence: the act of searching for information online can create an illusion of internal knowledge, where people begin to conflate access to the internet’s knowledge with their own (Fisher et al., 2015, 2022; Risko et al., 2016; Ward, 2021). This cognitive entanglement is a key mechanism of the ‘hollowed mind,’ as the metacognitive ability to distinguish between internal and external knowledge begins to decay (Ferguson et al., 2015; Fisher and Oppenheimer, 2021). A central risk of the ‘hollowed mind’ lies in the tendency to mistake access for ability.

A parallel erosion is well-documented in research on Global Positioning System (GPS) navigation. Studies show that while GPS is remarkably efficient, its over-reliance is correlated with poorer performance on spatial cognition tasks and a reduced ability to form accurate ‘cognitive maps’ of one’s environment (Dahmani and Bohbot, 2020; Hejtmánek et al., 2018; Ishikawa et al., 2008; Ruginski et al., 2019). Neuroimaging studies further indicate that the hippocampus—critical for spatial memory (Bird and Burgess, 2008; Burgess, 2008; Eichenbaum, 2017; Kunz et al., 2019)—shows reduced engagement during GPS-guided navigation (Maguire et al., 2000, 2003; Schinazi et al., 2013). The case of GPS reliance thus illustrates the hollowed mind, where successful task performance coincides with a progressive erosion of the underlying cognitive architecture.

### 3.2 Neuropsychological evidence: the ‘cognitive debt’ of AI use

Emerging neuroimaging and electrophysiological studies provide preliminary evidence that AI use alters the neural dynamics of cognition. Electroencephalography (EEG) research indicates that interaction with LLMs reduces frontal theta power, a well-established marker of working memory load (Jiang et al., 2025; Mai et al., 2021). While this may signal greater cognitive efficiency, it also suggests reduced engagement of the effortful processes central to learning. Complementary findings show increases in P300 amplitude—a component associated with attentional evaluation—during LLM use (Jiang et al., 2025), consistent with a shift from generative to evaluative modes of processing.

Preliminary evidence underscores both the promise and the risk of this neural reallocation. Kosmyna et al. (2025) reported up to a 55% reduction in cortical activity during AI-assisted writing, accompanied by impairments in subsequent memory integration. They describe this state as a form of “cognitive debt,” in which short-term efficiency is purchased at the expense of deeper encoding.

Together, these findings highlight a critical trade-off: AI support can reduce immediate cognitive burden, yet may undermine the very neural mechanisms required for durable knowledge formation—a dynamic also emphasized by (Dergaa et al., 2024). Given that AI systems themselves are still rapidly evolving, further longitudinal and replication studies are essential to clarify whether observed neural shifts reflect adaptive reallocation or detrimental disengagement.

### 3.3 The Expertise Duality: leveler and amplifier effects

The described risk of individual cognitive erosion through AI use appears to be at odds with the widely documented productivity benefits of generative AI (Brynjolfsson et al., 2024; Noy and Zhang, 2023; Paradis et al., 2025; Vaccaro et al., 2024; Ziegler et al., 2022). An emerging empirical consensus, however, reveals that this effect is highly conditional. A landmark 2024 meta-analysis of 106 experiments identified a single, critical determinant for successful human-AI collaboration: the human’s relative expertise. The study found that while teams often suffered from “negative synergy”—performing worse than their best member—this trend was inverted when the human was the more knowledgeable partner, leading to significant performance gains (Vaccaro et al., 2024). This dynamic is powerfully illustrated by large-scale field experiments. The Harvard-Boston Consulting Group (Harvard-BCG) study found that while AI leveled the performance of junior consultants, its use at the “jagged frontier”—where the AI was unreliable—caused novices to experience a 19-percentage-point performance drop, while experts who could identify and disregard flawed suggestions succeeded (Dell’Acqua et al., 2023). This pattern is corroborated across other domains, including software development and customer support, where gains are consistently concentrated among lower-skilled workers on routine tasks (Brynjolfsson et al., 2024; Noy and Zhang, 2023). The mechanism for this expert advantage appears to be increased cognitive effort: experts report investing more, not less, mental work when using AI to validate and integrate its outputs—the very work that novices bypass (Lee et al., 2025). The explanation for this apparent contradiction can be found in what we term the ‘Expertise Duality’: the impact of AI is contingent on the user’s prior knowledge and the complexity of the task at hand. Depending on the user’s expertise, AI takes on different roles, leading to a divergence in cognitive engagement:

1. The Leveler Effect (AI as an External Hippocampus for Novices): on routine tasks, AI provides structured guidance that helps novices achieve a level of performance they could not manage unaided (Wang and Fan, 2025). For a novice who lacks internal schemas, AI primarily functions as an external “hippocampal system,” providing the specific, episodic information needed to complete a task. However, the processes of validation, correction, and synthesis into new insights are likely to be omitted. This levels their performance but introduces a potential for dependency by bypassing neocortical learning.
2. The Amplifier Effect (AI as a Neocortical Partner for Experts): on complex, ambiguous tasks at the frontier of knowledge, AI only amplifies the capabilities of experts who possess the deep knowledge to direct the tool and validate its outputs. An expert possesses rich neocortical schemas. For them, AI can function as a “neocortical system” partner, rapidly generating patterns and data that their own well-developed internal models can then validate, correct, and synthesize into novel insights.

Depending on the user’s expertise and prior knowledge, AI takes on different roles. Different levels of prior knowledge among AI users thus lead to divergence in cognitive involvement in AI application, depending on their respective expertise. It is therefore plausible to suggest that this divergence will have a self-reinforcing effect with ubiquitous AI use and could thus exacerbate educational inequality. The fact that the general increase in economic productivity is currently attributable more to a leveling effect of AI through technological capabilities such as automation and speed is supported by two mechanisms: The first is the expertise reversal effect [as part of cognitive load theory (CLT, Kalyuga et al., 1998)], in which instructions that help beginners can overload and hinder experts (Armougum et al., 2020; Kalyuga, 2007; Kalyuga and Renkl, 2010; Mayer and Moreno, 2003). The second is the irony of automation, which describes how the automation of simple tasks paradoxically increases the cognitive load for experts who have to deal with complex edge cases and system failures (Bainbridge, 1983; Parasuraman et al., 2008; Parasuraman and Riley, 1997).

Both effects are thus also expressions of the principles of high effort for analytical and deliberative thinking described in Section 2. At the limits of existing knowledge and creativity, AI will therefore not significantly accelerate beyond the biopsychological determinants, as in the field of education. Ultimately, the threat of the “hollowed mind” is a direct functional consequence of this bypass. The leap from offloading calculation to offloading comprehension allows the user to skip the essential and effortful work of structuring arguments, reasoning through complexity, and synthesizing disparate information—the very processes proven to build durable memory and understanding (Bjork, 1994; Chi and Wylie, 2014; Slamecka and Graf, 1978). This systematic circumvention of deep cognitive work leads to a consequential confusion between instant access and genuine ability. The condition can thus be understood as a modern and more profound manifestation of automation-induced skill decay (Arthur et al., 1998; Macnamara et al., 2024), where the illusion of competence may mask a growing atrophy of our most critical thinking faculties. The underlying challenge arises from a core tension: genuine human cognition requires a slow, biological process of sustained engagement, a process that is difficult to reconcile with the affordance of instantaneous mastery.

## 4 The sovereignty trap: the psychological barrier to AI-assisted learning

The cognitive constraints outlined in the previous section give cause to a latent vulnerability in human learners. To describe how this vulnerability is exploited in an AI-saturated environment, we propose the ‘Sovereignty Trap’ as a theoretical principle. It arises from the collision of three forces: generative AI’s high-quality output, its frictionless availability, and the predictable human tendency to choose the path of least cognitive resistance—a trait well-documented as the law of least effort (Zipf, 1949), cognitive miserliness (Fiske and Taylor, 1984), and effort avoidance (Bocanegra et al., 2019; Kool et al., 2010). This tendency, however, is by no means uniform across individuals. It is profoundly shaped by what Cacioppo and Petty (1982) term the “Need for Cognition” (NfC)—an individual’s intrinsic motivation to engage in and enjoy effortful cognitive endeavors. Individuals with a high NfC actively seek out intellectual challenges and are more likely to engage their deliberate System 2, while individuals with a lower NfC, conversely, are motivated to avoid such effort and rely on heuristics and external cues. The Sovereignty Trap is therefore most potent for this latter group, as AI offers a perfect, socially acceptable release from the very cognitive strain they are predisposed to avoid. In such an environment, learners can be expected to preferentially opt for AI-generated solutions, even when aware that doing so undermines their long-term (learning) goals. While choosing ease is a rational decision in many contexts, the trap emerges in the domain of learning, where the most convenient choice is often the least beneficial, actively preventing the effortful cognitive engagement required to build durable knowledge. AI improves, this dynamic will only intensify, expanding the range of educational tasks solvable with its assistance to be near-universal.

The power of the Sovereignty Trap stems from a confluence of psychological mechanisms that reinforce one another, including:

- Cognitive Offloading and Process Bypass: Faced with a challenging task, there is a strong tendency to delegate effort to AI (Bocanegra et al., 2019; Risko and Gilbert, 2016). This can trigger automation bias (Mosier et al., 1998; Skitka et al., 1999; Zhai et al., 2024), leading to passive acceptance of outputs. This directly bypasses the germane cognitive load (Sweller, 1988) essential for schema construction. In doing so, it prevents the very cognitive processes that have been proven to create robust memories, such as self-explanation and retrieval practice, which are hallmarks of the most effective, “interactive” learning activities (Chi and Wylie, 2014).
- Effort Devaluation: Reliance on effortless answers devalues the knowledge itself. The ‘Effort Paradox’ shows that information acquired through struggle is not only valued more but is also encoded more robustly (Inzlicht et al., 2018). By providing frictionless solutions, AI inadvertently strips the learning process of the very effort that signals to the brain’s memory systems to prioritize encoding and consolidation, which can lead directly to “illusory competence” (Dell’Acqua et al., 2023).
- System 1 Dominance: AI’s frictionless outputs cater directly to our fast, intuitive System 1, which favors low-effort processing, while deep comprehension requires the deliberate, effortful System 2 (Kahneman, 2011). This systematically bypasses the cognitive engagement essential for retention. The constant availability of an ‘answer engine’ perpetually tempts users to rely on this heuristic processing, a dynamic that can be understood through the lens of ‘System 0’ (Chiriatti et al., 2024) which acts as a cognitive preprocessor, subtly steering users toward effortless information consumption and reinforcing System 1’s dominance. For millennia, the process of effective learning was inherently insulated by the simple absence of an easily accessible answer. AI removes this fundamental barrier at scale, reinforcing the very cognitive shortcuts education is meant to overcome.

Complementing this cognitive account, this dynamic can also be understood more profoundly through the motivational lens of Self-Determination Theory (SDT). From this perspective, the frictionless ‘answer engine’ appears to offer an ideal solution: it provides immediate answers, creating a powerful feeling of competence and satisfying the desire for quick task completion. This is the allure of the Sovereignty Trap. However, SDT posits that true, lasting motivation is fueled not by the illusion of competence, but by the satisfaction of the innate psychological need for earned competence and autonomy. In stark contrast, genuine learning—the very ‘struggle’ devalued by effortless access—is the process that fulfills this need. The Sovereignty Trap is therefore more than a cognitive trap; it is a motivational trap. It creates an environment where the path of least resistance systematically starves the learner of the intrinsic psychological rewards that fuel persistence, cultivate a growth mindset, and build a resilient identity as a capable agent. This suggests that the most effective AI scaffolds will need to not only structure cognitive tasks but also actively sense and support the learner’s motivational state.

### 4.1 Boundary conditions for strategic switching

The tendency to fall into the Sovereignty Trap, however, is not absolute. Humans are not passive dupes of technology; they can and do engage in strategic switching from default acceptance to active verification. Our framework posits that this crucial cognitive override is triggered by a set of key boundary conditions. Drawing on research in human-computer interaction and decision-making, these include:

- High Stakes and Accountability: When the consequences of an error are significant, or when a user knows they will be held accountable for their decision, the motivation to engage in effortful System 2 verification increases.
- AI Uncertainty Cues: When an AI explicitly signals its own uncertainty (e.g., by providing confidence scores or multiple, conflicting answers), it can prompt user vigilance and critical evaluation.
- Sufficient Prior Knowledge: As detailed in the Expertise Duality, users with a strong foundation of internal knowledge are far more likely to detect anomalies in an AI’s output, which serves as an internal trigger for verification.

The central argument of this paper is that the risk of the “hollowed mind” becomes most acute in environments where these boundary conditions are systematically absent—for instance, in low-stakes, formative learning contexts where AI outputs are superficially plausible and the learner lacks the prior knowledge to perceive any conflict.

Crucially, the Sovereignty Trap should not be considered as an indictment of AI itself. The emergence of sophisticated pedagogical tools, from Khan Academy’s Khanmigo to OpenAI’s Study Mode (2025) and Google’s LearnLM (2024), represents a positive and vital development. These systems are explicitly designed to offer stepwise scaffolding instead of instant answers. However, they remain fundamentally bypassable, as guided pedagogical systems are susceptible to being undermined by the parallel use of external ‘answer engines,’ allowing learners to create a deceptive illusion of engagement through the insertion of generated answers.

### 4.2 The normative value of cognitive sovereignty

The value of Cognitive Sovereignty is not absolute but context-dependent. We distinguish between two modes of AI use:

- Instrumental Use: in tasks where the primary goal is efficiency and accuracy for a discrete outcome (e.g., fixing boilerplate code), a calculated delegation of sovereignty can be entirely rational. Here, higher accuracy via delegation is often preferable to preserving agency.
- Integrative Use (Learning): in contexts where the primary goal is the construction of a user’s internal knowledge, preserving agency is paramount. Ceding sovereignty in this mode is counterproductive, as it undermines the very purpose of the activity.

This article focuses exclusively on the risks within these integrative learning contexts. While our analysis focuses on the individual learner, the principle of calibrated sovereignty generalizes to team and institutional levels, where the challenge becomes one of distributed cognition and shared epistemic responsibility.

## 5 Implications and imperatives for educational and system design

In this section, we outline the implications of the preceding analysis and formulate imperatives for future research and teaching in the field of AI & Education.

### 5.1 Navigating the cognitive trade-offs: key implications for education

1. Analytical and deliberative reasoning is a key competence in the age of AI. Simultaneously, AI itself poses the very risk of inducing cognitive atrophy and a “hollowed mind.”
2. Models from cognitive psychology converge on the finding that there is a fast and a slow system of cognitive processing. Deep learning is slow, effortful, and cannot be bypassed. The act of cognitive outsourcing to AI ought not to be viewed as a shortcut to knowledge; it is the direct removal of the learning process itself. Consequently, the application of AI in teaching and the design of AI literacy training must account for these fundamental psychological limitations.
3. The protracted developmental timeline of the prefrontal cortex, which extends beyond the teenage years, implies two critical vulnerabilities: first, teenagers and young adults are often not yet developmentally equipped to critically evaluate and reflect on AI-generated content. Second, during this crucial and sensitive period for higher-order learning, adolescents and young adults are particularly susceptible to AI-induced cognitive atrophy.
4. The very cognitive effort that AI promises to relieve us of may be precisely the effort our brains need in order to learn. The impact of AI on productivity depends on the individual user’s prior knowledge; for novices, this creates the risk of AI dependency, as neocortical learning is bypassed.
5. Given the differential impact of AI based on prior knowledge, the divergence in cognitive engagement during AI use could become self-reinforcing, leading to greater inequality among learners within a single group. This should be considered in the design of collaborative learning environments.
6. The instinctive default is to delegate effort to AI, which leads to the “Sovereignty Trap.” The development of illusory competence can distort a learner’s awareness of their own learning progress and knowledge acquisition. This tendency is not uniform across all learners. It is significantly moderated by individual differences in NfC. Learners with a low NfC are particularly vulnerable to the trap, as AI provides a perfect off-ramp from the cognitive strain they are predisposed to avoid.

### 5.2 A research agenda for the AI era

The psychology of AI in education is a nascent discipline, and the generation of longitudinal data to elucidate the mechanisms and dynamics specific to this field will require a considerable investment of time. It is imperative that established effects in the fields of learning and developmental psychology are re-examined and tested in the context of AI applications. Conversely, the adaptation of AI systems for educational purposes necessitates its own pedagogical design. A multi-faceted research agenda is hereby proposed, with its foundation resting upon three interwoven core areas: system design, psychological mechanisms, and socioeconomic implications.

#### 5.2.1 System design: from “answer engines” to cognitive training environments

The central design challenge is to transform AI from a tool that encourages cognitive bypass into one that fosters productive engagement. This requires moving beyond frictionless interfaces to create “choice architectures” for deep learning. Key research questions include:

##### 5.2.1.1 Operationalizing “desirable difficulties” through proven pedagogical patterns

How can we intentionally design AI interactions that introduce productive friction? Instead of inventing new methods from scratch, research must focus on empirically testing and implementing AI design patterns grounded in established pedagogical principles. This includes operationalizing Guidance Fading (Renkl and Atkinson, 2003), where AI support diminishes as user competence grows; Productive Failure (Kapur, 2016), where AI encourages exploration before providing solutions; and the ICAP framework (Chi and Wylie, 2014), designing interactions that push users from passive consumption towards active, constructive, and interactive engagement. The goal is to validate interfaces that transform the interaction from a passive query-response into an active, structured dialogue.

##### 5.2.1.2 Adaptive scaffolding based on real-time expertise assessment

The Expertise Duality demands systems that treat novices and experts differently. A crucial frontier is the development of AI tutors that can dynamically assess a user’s emerging expertise (e.g., through their prompts, revisions, or error patterns) and fluidly transition their support from “Leveler” (stepwise guidance) to “Amplifier” (a tool for complex synthesis), thereby avoiding the Expertise Reversal Effect.

##### 5.2.1.3 Building “metacognitive mirrors”

To combat the illusion of competence, how can AI systems make the user’s own cognitive processes visible to them? Research is needed on dashboards and feedback mechanisms that visualize the user’s degree of reliance on AI versus their own generative contributions, prompting self-reflection on their learning strategies and fostering genuine Cognitive Sovereignty.

#### 5.2.2 Psychological mechanisms: validating and deepening the “hollowed mind” framework

While our paper builds on established cognitive science, the unique nature of generative AI requires a new wave of empirical validation. The following questions are paramount:

##### 5.2.2.1 Longitudinal tracking of cognitive atrophy and fortification

The “hollowed mind” is a hypothesis about long-term change. Longitudinal studies are essential to track the cognitive and neural development of students who heavily rely on AI versus those who use it within structured, effortful frameworks. Do we see measurable skill decay in reasoning and synthesis, or does the brain adaptively reallocate its resources?

##### 5.2.2.2 Re-examining classic cognitive biases in the AI context

Foundational concepts like the Expertise Reversal Effect and the Ironies of Automation must be re-tested. Is the Expertise Reversal Effect more or less pronounced when the “instruction” is a fluid, conversational AI partner? Does the automation of reasoning (not just procedure) create new, more subtle forms of automation bias and skill decay? This research would update our core cognitive theories for the AI era.

##### 5.2.2.3 Operationalizing the sovereignty trap and strategic switching

How can we empirically measure both the act of ceding sovereignty and the decision to verify? Experimental designs should systematically manipulate the boundary conditions discussed in Section 4 (e.g., task stakes, accountability, AI uncertainty cues) and map their impact on measurable behavioral indicators, such as:

- Override rate on seeded errors (the frequency with which users correct deliberately flawed AI suggestions).
- Verification latency (the time and effort users invest in cross-checking AI outputs).
- Quality of evidence sought during verification (e.g., consulting high- vs. low-quality external sources).

##### 5.2.2.4 Investigating individual differences as moderators

The risk of the “hollowed mind” is likely not uniform. Future research must investigate how stable cognitive traits moderate the effects of AI use. For instance, are individuals with a low NfC (Cacioppo and Petty, 1982) or poor performance on the Cognitive Reflection Test (Frederick, 2005) significantly more vulnerable to the Sovereignty Trap and subsequent skill decay? Identifying such at-risk populations is a critical step toward developing targeted pedagogical interventions.

#### 5.2.3 Economic and sociological implications: the macro-level consequences

The interaction between individual cognition and AI scales up to produce societal-level effects that require urgent investigation.

##### 5.2.3.1 The great equalizer or the great divider?

The Expertise Duality has profound economic implications. Will the “Leveler” effect on novices reduce skills gaps and promote equity? Or will the “Amplifier” effect allow top experts to achieve unprecedented productivity, creating a new “super-expert” class and widening socioeconomic inequality? Economic modeling and large-scale field studies are needed to understand AI’s true impact on the labor market’s skill distribution.

##### 5.2.3.2 Redefining and assessing 21st-century competence

If AI automates routine knowledge work, what constitutes a valuable education? Research must focus on developing new assessment methods that can validly measure “Cognitive Sovereignty,” metacognitive resilience, and the ability to manage AI as a tool—the core competencies of the “fortified mind.” How do educational institutions and employers shift from evaluating what a person knows to evaluating how they think in an AI-saturated environment?

##### 5.2.3.3 The future of epistemic authority

In a world flooded with high-quality, AI-generated content, how do individuals and societies maintain a shared sense of truth? Sociological and communication studies are needed to understand how AI is changing our relationship with traditional sources of epistemic authority (e.g., science, journalism, education) and what new literacies are required to navigate this complex new information ecosystem.

### 5.3 Cultivating the fortified mind: an educational imperative

The necessary antidote to the risks of the “hollowed mind” ought not to be viewed as the rejection of AI, but the deliberate cultivation of what we term the “Fortified Mind.” This concept moves beyond a simple accumulation of facts to describe a resilient and dynamic and adaptive cognitive architecture, specifically built to thrive in an AI-saturated environment. This internal architecture should not be conceived as a static defense, but as a durable yet plastic foundation for lifelong adaptation and growth. It is a multi-dimensional construct composed of three integrated and mutually reinforcing layers. This construct explicitly excludes the simple accumulation of isolated, easily retrievable facts, focusing instead on transferable structures of thought. First, it is built upon a foundation of robust, domain-specific schemas (Sweller, 1988). This internal knowledge should not be conflated with a static library for validating AI outputs; it is the essential substrate for the human mind’s unique strength as a dynamic association machine. It is the deep, transferable knowledge of a domain’s core principles that allows an individual to recognize patterns, understand context, and identify when an AI’s output is plausible but fundamentally flawed. Second, it possesses well-practiced metacognitive skills (Flavell, 1979). This is the capacity for self-monitoring and epistemic vigilance—the ability to ask critical questions not just of the AI, but of oneself: “How do I know this is correct? Am I outsourcing judgment out of convenience? What are the limits of my own understanding here?” Finally, and perhaps most crucially, the Fortified Mind is characterized by the disposition for effortful System 2 thinking (Kahneman, 2011). It is not merely the ability to think deeply, but the cultivated habit of engaging in the “desirable difficulties” (Bjork, 1994) that are essential for genuine learning. This internal architecture is the functional prerequisite for achieving Cognitive Sovereignty. It is what enables a user to escape the Sovereignty Trap by providing the internal reference points needed to challenge and overrule an AI. Furthermore, it is the very engine that resolves the Expertise Duality, transforming AI from a mere Leveler into a true Amplifier of human intellect. The cultivation of this internal fortress, therefore, becomes the primary imperative for educational and system design, providing the guiding principle for the implications and research agenda set out above.

## 6 Conclusion and outlook

This paper has examined the central duality at the heart of generative AI in education: its dual capacity to act as a powerful cognitive extender while posing a significant risk of cognitive atrophy. We have argued that the dominant “answer engine” paradigm, while optimized for immediate efficiency, is in tension with the biological and psychological constraints of human learning. By systematically enabling a “cognitive bypass” of the effortful processes required for knowledge internalization, this design philosophy creates a significant risk of fostering what we term a “hollowed mind”—a cognitive state where access to information is mistaken for genuine ability. Our analysis, grounded in a synthesis of cognitive science, neurobiology, and developmental psychology, has sought to substantiate this risk. We identified the Sovereignty Trap as a key psychological mechanism that may tempt learners to cede their intellectual agency in favor of frictionless, authoritative AI outputs. We demonstrated how this dynamic appears not to be uniform, but manifests as the Expertise Duality, where AI levels the performance of novices while amplifying the capabilities of experts, potentially widening rather than closing educational and economic divides. The central conclusion of this paper is therefore not a rejection of technology, but a reaffirmation of a core pedagogical principle, grounded in biological evidence: that the slow, effortful process of building internal knowledge remains indispensable. This reframes the learner’s question—“Why strive for expertise when an AI is superior?” Expertise can be understood not as a competing repository of facts, but as the very cognitive architecture required to effectively partner with, govern, and create value from AI. The “fortified mind,” in this view, is not an alternative to AI; it is its essential prerequisite. Harnessing the potential of AI for education, therefore, calls for a new design philosophy—one that treats human cognitive limitations not as bugs to be engineered away, but as features to be designed for. This means building systems that scaffold, challenge, and reflect, rather than merely respond. This presents a considerable challenge, as it requires the design of tools that work in concert with our cognitive nature, even when that nature pushes us toward the path of least resistance. Ultimately, this perspective suggests that the promise of AI in education is not to render knowledge obsolete, but to highlight the pursuit of it as more vital than ever—transforming it from a static asset into the dynamic foundation of human Cognitive Sovereignty.

## 7 Key definitions and theoretical constructs

### 7.1 Cognitive sovereignty

The enacted behavior of exercising intellectual independence in AI-rich environments. It manifests as the ability to set goals, validate outputs, and appropriately overrule the tool when necessary. Cognitive Sovereignty excludes mere influence; being persuaded by a well-reasoned argument (from a human or AI) is not a loss of sovereignty. The loss occurs only with uncritical acceptance or the inability to reconstruct and validate the justificatory chain of the output. The ideal state is not absolute independence, but ‘calibrated sovereignty’: a rational and permissible reliance on tools or other agents, contingent on the user’s ability to maintain transparency and revisability over the justificatory chains. Related to: Metacognition (Flavell, 1979), Epistemic Agency, Expertise Reversal Effect (Kalyuga et al., 2007).

### 7.2 Sovereignty trap

The psychological trap where an AI’s authoritative competence and frictionless output tempts a user to cede their own intellectual sovereignty—their role as the ultimate arbiter of truth, relevance, and purpose in their cognitive work. Related to: Cognitive Miserliness (Fiske and Taylor, 1984), Law of Least Effort (Zipf, 1949), Automation Bias (Mosier et al., 1998).

### 7.3 Fortified mind

A multi-dimensional cognitive architecture that enables Cognitive Sovereignty in AI-rich environments. It is characterized by three core components:

1. a well-organized base of domain-specific schemas (in line with Schema Theory).
2. proficient metacognitive skills for self-monitoring and epistemic vigilance (e.g., knowing when to verify AI outputs).
3. an ingrained disposition for effortful System 2 thinking.

This construct explicitly excludes isolated, easily retrievable facts and focuses instead on transferable mental models and reasoning processes. Related to: Expertise and Schema Theory (Sweller, 1988), Desirable Difficulties (Bjork, 1994).

### 7.4 Hollowed mind

A cognitive state characterized by superficial knowledge and dependency on external tools, resulting from the chronic bypassing of deep processing. Related to: Cognitive Offloading (Risko and Gilbert, 2016), Automation-Induced Skill Decay (Arthur et al., 1998).

### 7.5 Cognitive bypass

The mechanism by which an AI system, designed for the frictionless delivery of plausible answers, enables a user to circumvent the effortful cognitive processes (e.g., conflict detection, deliberative reasoning, and schema construction) that are foundational to genuine learning and knowledge internalization. Related to: Cognitive Miserliness (Stanovich, 2011), Germane Load (Sweller, 1988), Override Mechanism (Evans and Stanovich, 2013), Cognitive Reflection (Frederick, 2005).

### 7.6 Conceptual typology of the hollowed mind

To provide a clear and testable framework, we distinguish between the cognitive state, the underlying mechanisms, and the observable symptoms associated with the risks of AI use.

*State*: The Hollowed Mind. We define the ‘hollowed mind’ as a long-term, stable cognitive state characterized by dependency and diminished capacity for deep reasoning. This is the ultimate outcome of chronic under-engagement.

- This state can be operationalized by measuring longitudinal skill decay, such as poor performance on novel transfer tasks or complex problem-solving scenarios performed *without* AI assistance.

*Mechanisms*: Cognitive Bypass & Sovereignty Trap. We define these as the short-term, repeated psychological processes that, over time, can lead to the ‘hollowed mind’ state. The Cognitive Bypass is the cognitive mechanism of circumventing effortful processing. The Sovereignty Trap is the motivational mechanism of ceding intellectual judgment.

- The Cognitive Bypass can be measured in real-time by observing a bypass of germane cognitive load, for example, through reduced time-on-task, lower frontal theta power in EEG, or fewer self-explanation prompts.
- The Sovereignty Trap can be operationalized by measuring a user’s susceptibility to automation bias, such as the uncritical acceptance rate of flawed AI suggestions.

*Symptoms and precursors*: These are the immediate, observable behaviors and related phenomena that indicate the mechanisms are at play.

Antecedent user traits (e.g., prior knowledge, need for Cognition) contribute to the development of the Fortified Mind (the internal capacity), which in turn enables the enactment of Cognitive Sovereignty (the observable behavior). The chronic failure to enact this behavior, through the mechanisms of the Cognitive Bypass and Sovereignty Trap, can eventually lead to the degradation of the underlying capacity, resulting in the Hollowed Mind state.

## Data Availability

The original contributions presented in the study are included in the article/supplementary material, further inquiries can be directed to the corresponding author.
